# Managing Antidepressant‐Induced Hyperhidrosis With Vitamin E: An Over‐the‐Counter Supplement–Based Approach

**DOI:** 10.1002/ccr3.71979

**Published:** 2026-03-11

**Authors:** Saad Nazir, Mahnoor Azmat, Neha Prathivadi, Divyaraj Bavishi, Yasin Ibrahim

**Affiliations:** ^1^ Department of Psychiatry University of Texas at Tyler Tyler Texas USA; ^2^ Department of Psychiatry Texas Tech University Health Sciences Center Lubbock Texas USA

## Abstract

Excessive sweating is a common side effect of antidepressants, affecting approximately 22% of patients. Antidepressant‐induced excessive sweating (ADIES) can significantly impair quality of life and medication adherence. This case report explores the use of Vitamin E as a novel adjunct treatment for ADIES. A woman in her 30s with major depressive disorder experienced significant improvement in depressive symptoms with venlafaxine but developed distressing night sweats at higher doses. Given the patient's reluctance to switch to a different antidepressant and to reduce polypharmacy, a trial of over‐the‐counter Vitamin E was initiated based on its prior evidence in managing hot flashes in postmenopausal women. After 3 months, the patient reported a significant reduction in sweating, as measured by validated symptom severity scales (CGI‐S and HDSS). The case highlights Vitamin E as a potentially effective, low‐risk, and accessible option for ADIES, especially for patients at risk for polypharmacy. While larger studies are needed, Vitamin E may offer a simple strategy for improving quality of life and medication adherence.

## Key Clinical Message

Vitamin E, an accessible antioxidant supplement, may reduce antidepressant‐induced hyperhidrosis when medication changes are not acceptable. The case highlights continued use of an antidepressant while mitigating the side effect of hyperhidrosis with the use of Vitamin E. Improvement indicated temporal association but may reflect placebo effects or regression to the mean.

## 1 Background

Antidepressant‐induced excessive sweating (ADIES), often referred to as “night sweats,” is a well‐documented adverse effect associated with most classes of antidepressants. This side effect not only causes significant discomfort but also impacts treatment adherence. Approximately 22% of patients on antidepressants experience ADIES [1]. Venlafaxine, a serotonin‐norepinephrine reuptake inhibitor (SNRI), is particularly noted for this effect. It is hypothesized that the increase in sweating seen at higher doses may be due to an increasing noradrenergic active component [2, 3]. Despite its high prevalence, ADIES remains under‐studied, with limited data on effective treatments. This report presents a case in which Vitamin E supplementation demonstrated efficacy in alleviating ADIES symptoms.

## 2 Case Presentation

A woman in her 30s presented with a history of Major Depressive Disorder and Persistent Depressive Disorder to the outpatient clinic for an initial visit. She endorsed symptoms including anhedonia, low energy, disturbed sleep with nightmares, impaired concentration, hopelessness, and guilt. She reported long‐standing depression and suicidal ideation beginning in adolescence and had trialed several SSRIs over the years without significant improvement. She denied a history of mania while on SSRIs. Her symptoms were primarily depressive rather than anxiety‐driven. She had a history of early alcohol use but had abstained since her pregnancy in her twenties. She denied any active substance use during that initial visit. She had never been hospitalized in a psychiatric facility nor previously evaluated by a psychiatrist. Her medical history was notable only for hypertension. There was no reported history of thyroid abnormalities, OCP use, seizures or head trauma and family history was significant for bipolar disorder in her grandmother. She denied symptoms of psychosis, PTSD, OCD, or social anxiety.

Given her lack of response to SSRIs, an SNRI venlafaxine was initiated at 75 mg and titrated to 150 mg daily in the morning over 4 weeks. The patient reported marked symptom improvement at the 3 month follow‐up visit, including improved energy and mood, with ~75% symptom resolution as described in the patient's own words albeit with reports of new onset mild sweating which was not bothersome. Two months later, the patient reported worsening of depression in the context of psychosocial stressors. The dose was increased to 187.5 mg daily to address symptom recurrence. Her symptoms significantly improved with the increased dose; however, the previously reported mild sweating had now worsened to a point that it interfered with sleep and daily functioning, which the patient reported finding “frustrating”. Despite this side effect, the patient declined dose reduction, switching medications, or adding additional medications. Subsequently, Vitamin E was proposed as a supplement‐based option based on previous evidence in improving sweating and hot flashes in postmenopausal women. The dose of venlafaxine remained unchanged at 187.5 mg/day with no new psychotropic medications during the trial of Vitamin E.

## 3 Investigation

Investigations including complete blood count, comprehensive metabolic panel and thyroid studies were within normal limits. No specific investigations were required for monitoring in this case. The clinical picture was primarily informed by symptom reporting and validated symptom rating scales (CGI‐S and HDSS), which were instrumental in assessing the efficacy of the intervention.

## 4 Treatment

Prior to initiation of Vitamin E it is ideal to screen the patients for bleeding risk (use of anti‐coagulants) and counsel them about potential bruising or mucosal bleeding. Routine laboratory monitoring is not required, but clinical monitoring is usually sufficient. In this case, the patient reported no adverse effects with Vitamin E, including any changes in her blood pressure, sleep, or energy.

Supplement‐based interventions, including room temperature control, hydration, and breathable clothing, were initially suggested but proved ineffective. The patient then agreed to initiate Vitamin E at 134 mg (200 IU) daily. This dose was selected in lieu of pursuing the minimum effective dose strategy. The patient reported symptom improvement within 1 week, which was sustained over several months.

Vitamin E formulation was d‐alpha‐tocopherol 200 IU (≈134 mg) softgel once daily. This starting dose was selected for its favorable tolerability profile and prior use in vasomotor symptom trials; step‐up titration was intentionally avoided to minimize pill burden while assessing response.

## 5 Outcome and Follow‐Up

Prior to the Vitamin E treatment, the patient described her symptoms as daily, continuous sweating with worsening during the nighttime resulting in disturbed sleep with 3–5 awakenings during the night.

Patient completed three validated scales—the CGI Severity Scale (CGI‐S), CGI Improvement Scale (CGI‐I), and the Hyperhidrosis Disease Severity Scale (HDSS) [4, 5, 6]. The CGI‐S and CGI‐I are used to evaluate the severity and improvement in the patient's illness, respectively. HDSS measures the level of hyperhidrosis and degree of interference with daily living. She rated her symptoms as 6/7 (severely ill) on the CGI‐S and 4/4 (intolerable, always interferes with daily activities) on the HDSS prior to Vitamin E treatment as shown in Table 1.

**TABLE 1:** Self‐reports of three validated symptom severity scales. Scales used: Clinical Global Impression—Severity (CGI‐S), Clinical Global Impression—Improvement (CGI‐I), and Hyperhidrosis Disease Severity Scale (HDSS) [4, 5, 6].

Scale	Pre‐treatment	Post treatment
CGI‐S (out of 7)	6—severely ill	2—borderline mentally ill
CGI‐I (out of 7)	Not applicable	2—much improved
HDSS (out of 4)	4—intolerable, interferes with daily activities	2—tolerable, sometimes interferes with daily activities

After 3 months of Vitamin E, she reported episodic sweating (1–2 days/week), with night awakenings only a few times per month. Post‐treatment, she rated her CGI‐S as 2/7 (borderline mentally ill), CGI‐I as 2/7 (much improved), and HDSS as 2/4 (tolerable, sometimes interferes with daily activities) as shown in Table 1.

## 6 Discussion

### 6.1 Interpretation Caveats

The observed improvement represents a temporal association. Potential confounders include seasonal variation, spontaneous fluctuation with regression to the mean, and placebo effects. These considerations are now noted to contextualize the response and avoid over‐attribution to Vitamin E alone.

Antidepressant‐induced excessive sweating (ADIES), sometimes colloquially referred to as “night sweats,” is a common complaint from patients taking antidepressants of any class. Approximately 22% of patients taking antidepressants experience symptoms of ADIES [1]. While specific antidepressants or antidepressant classes have not been identified to be more likely to cause hyperhidrosis when compared to others, bupropion, fluvoxamine, and vortioxetine are three antidepressants that may have a decreased risk of causing hyperhidrosis [2].

Venlafaxine is particularly implicated, likely due to increased sympathetic and noradrenergic activity, increasing the production of sweat. The symptoms of sweating can worsen with increasing doses of Venlafaxine [3].

The discomfort of ADIES can negatively impact medication adherence. Various medications have been used to treat ADIES, including terazosin, cyproheptadine, oxybutynin, and clonidine [1]. While these medications have been used with some success, evidence remains limited, and no treatment has emerged as clearly superior. The clinical implication of treating a side effect of one medication with another medication raises the concern for polypharmacy. Recently, Vitamin E, a widely available supplement, has gained interest as a potential treatment option to address ADIES while mitigating the risk of polypharmacy. The potential for Vitamin E as a treatment option for hyperhidrosis associated with ADIES is derived from data showing Vitamin E to be effective for postmenopausal women experiencing hot flashes. Multiple studies have shown that Vitamin E significantly reduced hot flashes for postmenopausal women and breast cancer survivors [7, 8, 9, 10]. It is important to recognize that an excess of Vitamin E may be associated with increased mortality, so there is a need to find a balance with the patient that maximizes the benefits with minimal risk [4].

Potential advantages of using Vitamin E include that the supplement is nonhormonal, is inexpensive and widely available, limits the concern for polypharmacy, and is safe at moderate levels of usage. Past literature supports the use of Vitamin E as a safe and mildly beneficial option for patients with hot flashes and sweats, and is worth trying especially for patients on multiple other medications or who are exploring non hormonal options [or in those that traditional meds may be contraindicated such as the elderly].

## 7 Learning Points/Take Home Messages

– Excessive sweating is a common side effect of antidepressants, which can significantly impair quality of life and medication adherence.
– Vitamin E may represent a safer supplement over‐the‐counter alternative to traditional treatments for ADIES such as terazosin and prazosin.
– Vitamin E may be considered in patients when there is a concern for postural hypotension or anticholinergic side effects of traditional treatments such as the elderly, and may be especially effective in post‐menopausal women with concurrent complaints of hot flashes, as evidenced by studies [2].

## Author Contributions

**Saad Nazir:** conceptualization, formal analysis, supervision, writing – original draft, writing – review and editing. **Mahnoor Azmat:** writing – original draft, writing – review and editing. **Neha Prathivadi:** writing – original draft. **Divyaraj Bavishi:** writing – review and editing. **Yasin Ibrahim:** writing – review and editing.

## Funding

The authors have nothing to report.

## Disclosure

The lead author affirms that this manuscript is an honest, accurate, and transparent account of the case being reported. No important aspects have been omitted, and all relevant context has been included.

## Ethics Statement

The authors assert that all procedures contributing to this work comply with the ethical standards of the relevant national and institutional committees on human experimentation and with the Helsinki Declaration of 1975, as revised in 2013. As this is a single‐patient case report with no experimental intervention, ethics committee approval was not required.

## Consent

Written informed consent was obtained from the patient for the publication of this case report. The patient was fully anonymized, and no identifiable information is included in the manuscript.

## Conflicts of Interest

The authors declare no conflicts of interest.

## Data Availability

Data availability is not applicable to this article as no new datasets were generated or analyzed during this case report.
